# Assessment of adolescent idiopathic scoliosis from body scanner image by finite element simulations

**DOI:** 10.1371/journal.pone.0243736

**Published:** 2021-02-10

**Authors:** Alexander T. D. Grünwald, Susmita Roy, Ana Alves-Pinto, Renée Lampe

**Affiliations:** 1 Orthopaedic Department, Klinikum rechts der Isar, School of Medicine, Technical University of Munich, Munich, Germany; 2 Markus Würth Professorship, Technical University of Munich, Munich, Germany; University of California San Francisco, UNITED STATES

## Abstract

Adolescent idiopathic scoliosis, is a three-dimensional spinal deformity characterized by lateral curvature and axial rotation around the vertical body axis of the spine, the cause of which is yet unknown. The fast progression entails regular clinical monitoring, including X-rays. Here we present an approach to evaluate scoliosis from the three-dimensional image of a patient’s torso, captured by an ionizing radiation free body scanner, in combination with a model of the ribcage and spine. A skeletal structure of the ribcage and vertebral column was modelled with computer aided designed software and was used as an initial structure for macroscopic finite element method simulations. The basic vertebral column model was created for an adult female in an upright position. The model was then used to simulate the patient specific scoliotic spine configurations. The simulations showed that a lateral translation of a vertebral body results in an effective axial rotation and could reproduce the spinal curvatures. The combined method of three-dimensional body scan and finite element model simulations thus provide quantitative anatomical information about the position, rotation and inclination of the thoracic and lumbar vertebrae within a three-dimensional torso. Furthermore, the simulations showed unequal distributions of stress and strain profiles across the intervertebral discs, due to their distortions, which might help to further understand the pathogenesis of scoliosis.

## 1 Introduction

One of the most common reasons for an appointment with paediatric and neuro-orthopaedic specialists are idiopathic and neuromuscular scoliosis. Scoliosis is a medical condition characterized by a three-dimensional (3D) deformity of the vertebral column, due to lateral curvatures of the spine of at least 10° and rotations and torsions in the transverse plane around the vertical body axis [1]. Idiopathic scoliosis is the most common type of scoliosis in children between 10 and 18 years of age [2, 3]. It is a complex medical condition with very different forms of appearance and multifactorial aetiology that is not yet fully understood. Early detection and regular monitoring of idiopathic scoliosis are thus very important to prevent progression and to improve the malformation of the vertebral column. One of the very first clinical approaches in the assessment of scoliosis is the Adam’s forward bend test, where the height difference of the back surfaces to either side of the spine is measured [4]. In addition to the clinical examination, the severity of scoliosis is generally quantified by the Cobb angle [5], which requires a posterior-anterior radiograph of the spine. In consequence of frequent radiographic examinations, young patients diagnosed with scoliosis have a high risk of ionizing radiation related future health problems [6]. In order to minimize the radiation exposure to patients, several clinical examination methods have been developed to detect and evaluate scoliosis.

Surface topography methods are broadly used in monitoring of scoliosis, by scanning the torso surface with non-invasive, visible light. The different imaging techniques, such as Moiré-fringe mapping [7, 8], rasterstereography [9], integrated shape imaging system (ISIS) [10, 11], the quantec system [12, 13] and laser triangulation [14], are used to estimate the alignment of the spine. These techniques mainly capture geometrical and morphological changes in the torso surface from the surface topography and from asymmetries of the torso shape. The studies, however, yield different results when determining spinal deformity indices of scoliosis [15, 16]. These mismatches in the results are partly due to technical limitations of the imaging systems, or other physical issues, which are not related to scoliosis, such as normal growth, posture, body fat and others [16]. Surface topography based methods are currently the most commonly used ionizing radiation free techniques in clinical practice, in order to minimize the risk of radiation related secondary diseases from multiple X-rays.

In scoliosis the axial rotation around the vertical body axis of the vertebrae is correlated with the lateral deviation, which has a significant effect on the initiation and progression of scoliosis. The reason for these lateral translation and rotation, however, is not clear yet. For this purpose and with the availability of increased computational power, several mathematical and biomechanical models of the human spine have been developed and studied [17–20]. Finite element method (FEM) is a widely used simulation tool for the understanding of microscopic and macroscopic structural changes of the spine and its components, associated with scoliosis. For example, FEM based biomechanical models of the thorax and isolated ligamentous spine models were constructed by Stokes et al. in order to investigate the aetiology of scoliosis [17, 21]. The results support the hypothesis that an asymmetric growth of the thorax could initiate a small lateral curvature and axial rotation of the spine during adolescence [21]. In another study, Drevelle et al. investigated the biomechanical factors involved in the mechanism of the progression of idiopathic scoliosis, by FEM simulations. They studied the effect of gravity and anterior spinal overgrowth on vertebral axial rotation and lateral deviation in case of progressing scoliosis [18]. Besides that, the 3D biomechanical model of the idiopathic scoliosis patients’ spine was simulated by using FEM, for the strategy planning of scoliosis surgery [19].

In principle, FEM based modelling enables a wide range of research possibilities. Instead of laboratory experiments, different physiological and anatomical conditions can be simulated to understand biomechanics, such as a scoliotic vertebral column. Depending on the research questions and purpose, the vertebral column model can be created from simple to complex. For example, a simplified three-dimensional spine model was created by Dietrich et al. [22] to estimate the elemental forces and stresses at different positions of the spine. In contrast a more complex three-dimensional finite element model, including ribcage, thoraco-lumbar spine, pelvis and soft tissues was created to simulate the spinal response resulting from a specific manipulative force, applied to the lumbar spine [23]. Thereby the individual effects of various components of the vertebral column on the spinal response upon applied forces can be investigated without invasive techniques. More detailed and hence complex models of the vertebral column, including elaborated component geometries and anatomical details of each component, such as vertebral bodies [24, 25], intervertebral discs (IVDs) [26], muscular and ligament elements [27], were made for detailed biomechanical investigations of the human spine [20].

In the present study, a basic skeletal structure of the essential components of the ribcage and vertebral column was modelled with computer aided design (CAD) software and was used as initial structure for macroscopic FEM simulations. In a first approach the model was simplified to the essential components, considering the vertebrae, the IVDs, the ribs and the sternum. The IVDs and ribs were considered as anisotropic elastic materials. The bony vertebral bodies and the sternum were regarded as rigid bodies. The initial geometry of the model was set according to the anatomical specifications of a female adult, with the option to change for a male configuration. The developed method allowed us to generate, modify and investigate a variety of patient specific spinal configurations at different vertebral levels and ribcage geometries, within certain limits.

With respect to future clinical applications, the present method was designed for the analysis of 3D images of the patients torso collected with a camera system, hereinafter referred to as body scanner. The body scanner was custom designed for the assessment of scoliosis, especially in young patients, and therefore does not use ionizing radiation. The present simulations were performed to analyse the 3D scan images of two teenage females, captured with the body scanner, who have been diagnosed with adolescent idiopathic scoliosis. Generally, for a patient with spinal deformity, an X-ray image is an essential standard of care at the first appointment with an orthopaedic specialist. For the method presented here, in addition a body scan was taken at the same day. Both images were then used as target for the ribcage and vertebral column model simulations. In order to obtain a good match of the spinal curvature with the X-ray and transverse body scanner images, specific parameters of the current model had to be adjusted.

In the present study we have also analysed the interrelation between the lateral deviation of the vertebrae and their axial rotation around the vertical body axis upon a predefined translation and rotation applied to certain vertebral levels. In addition the distributions of stress and strain on the IVDs, associated with the deviation, were studied from the FEM calculations in order to gain further understanding of the biomechanical pathogenesis of scoliosis, in terms of stress and strain. Furthermore, the present methods can help an orthopaedic specialist to get anatomical related information about the position and axial rotation of the vertebrae and ribcage from the 3D surface scan image. The combination of body scanner images and FEM model simulations thus have the potential to reduce the number of X-rays in follow up examinations, in complement to clinical investigations.

## 2 Materials and methods

### 2.1 Experimental data

**Fig 1:**
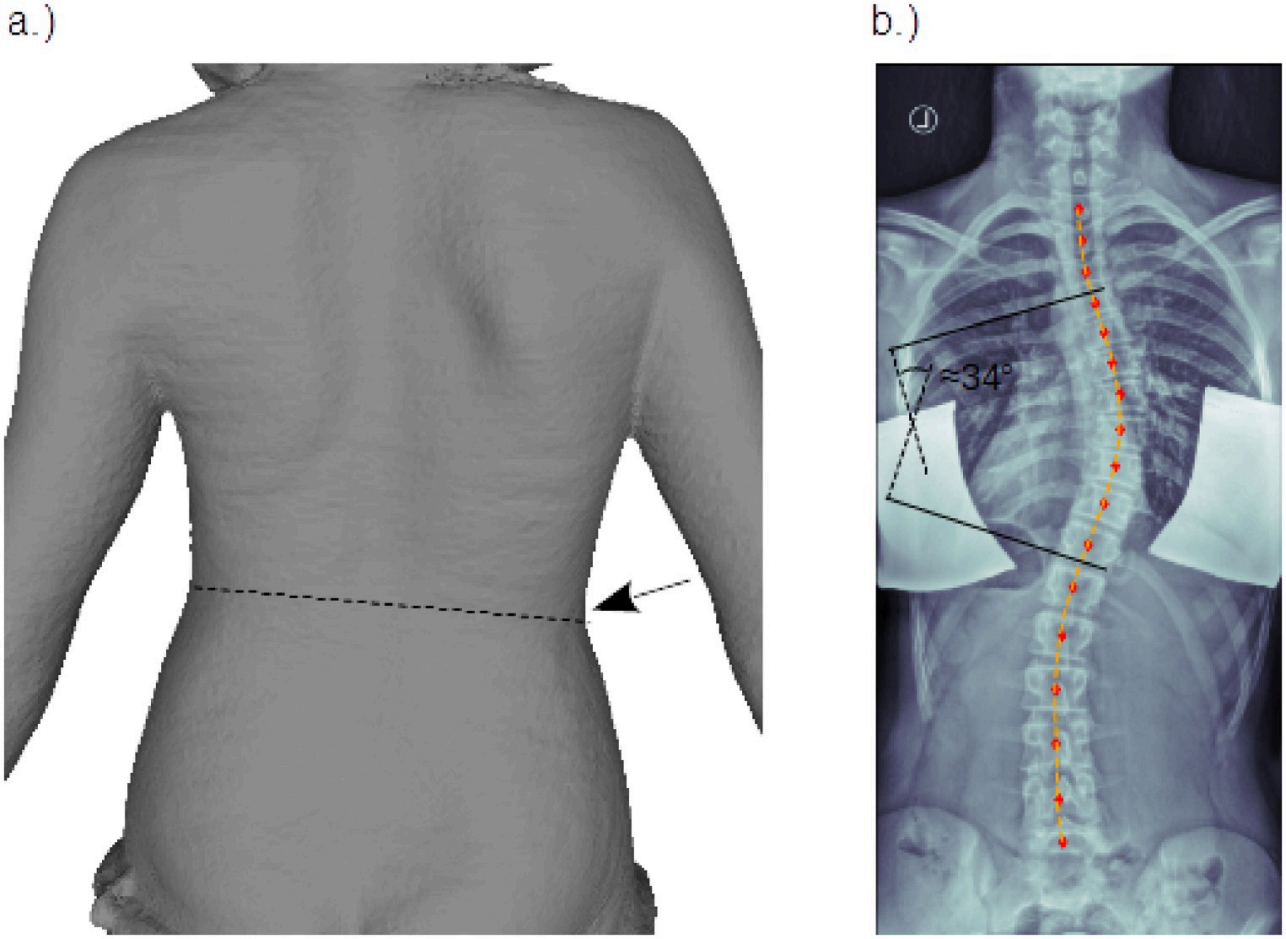
Patient images. Posterior-anterior view of a female adolescent with thoracic right convex scoliosis: a.) body scanner image, with slightly inclined waistline (dashed line) and more pronounced waist on the right side, marked by arrow. b.) X-ray image (left/right flipped) with Cobb angle annotation. The red markers indicate the center positions of the vertebral bodies, using analysis tools described in [29]. The orange dashed line shows the result of a least squares polynomial fit through the marker positions. This fit, hence describes the course of the vertebral column in the coronal plane and is then further used for comparison with the model simulations.

Here we analysed the body scanner and X-ray images of two adolescent females. Patient one (P1) was about 14 years old and diagnosed with a thoracic right convex scoliosis with its apex around level T8, a Cobb angle of 34° and a rotational degree of Moe I. In terms of the Lenke classification system [28] her scoliosis was described as main thoracic curve—type 1—with lumbar spine modifier A. In addition a small asymmetry of the waistline with a more pronounced waist on the right side was found in standing upright position, also visible in the rear view of the body scanner image, Fig 1a. The corresponding X-ray image is shown in Fig 1b. Patient two (P2) was 16 years old and diagnosed with a double major curve—type 3—and a lumbar spine modifier A, according to the Lenke classification system, a thoracic Cobb angle of 46°, a lumbar Cobb angle of 33° and a rotational degree of Moe II. To show our working principle, the body scanner and X-ray images of P1 only are presented as an example.

The 3D surface scan images of the patient’s torso were taken with a custom-designed body scanner system at the orthopaedics department at the Klinikum rechts der Isar of the Technical University of Munich. The body scanner system is based on a RGB-D sensor, mounted on a scan arm that rotates in the transverse plane around the standing, or sitting, patient within less than fifteen seconds and provides a conformal 3D surface scan image of the outer body shape contour [29]. The system is thus non-invasive and works without ionizing radiation.

The present project and all its procedures related to patients data privacy and personal interests were approved in advance by the ethics committee of the Technical University of Munich, Faculty of Medicine in Munich, Germany (Ref 569/16 S).

### 2.2 Modeling data

A bony model of the average human ribcage was constructed with CAD, using FreeCAD—an open-source parametric 3D modeler made to design real-life objects [30]. This model was then used in FEM calculations to simulate the subject specific deformations of the ribcage and vertebral column. In the following, the characteristics of the model and its individual components are described in more details.

#### 2.2.1 Model of ribcage and vertebral column (MoRCaVC)

**Fig 2:**
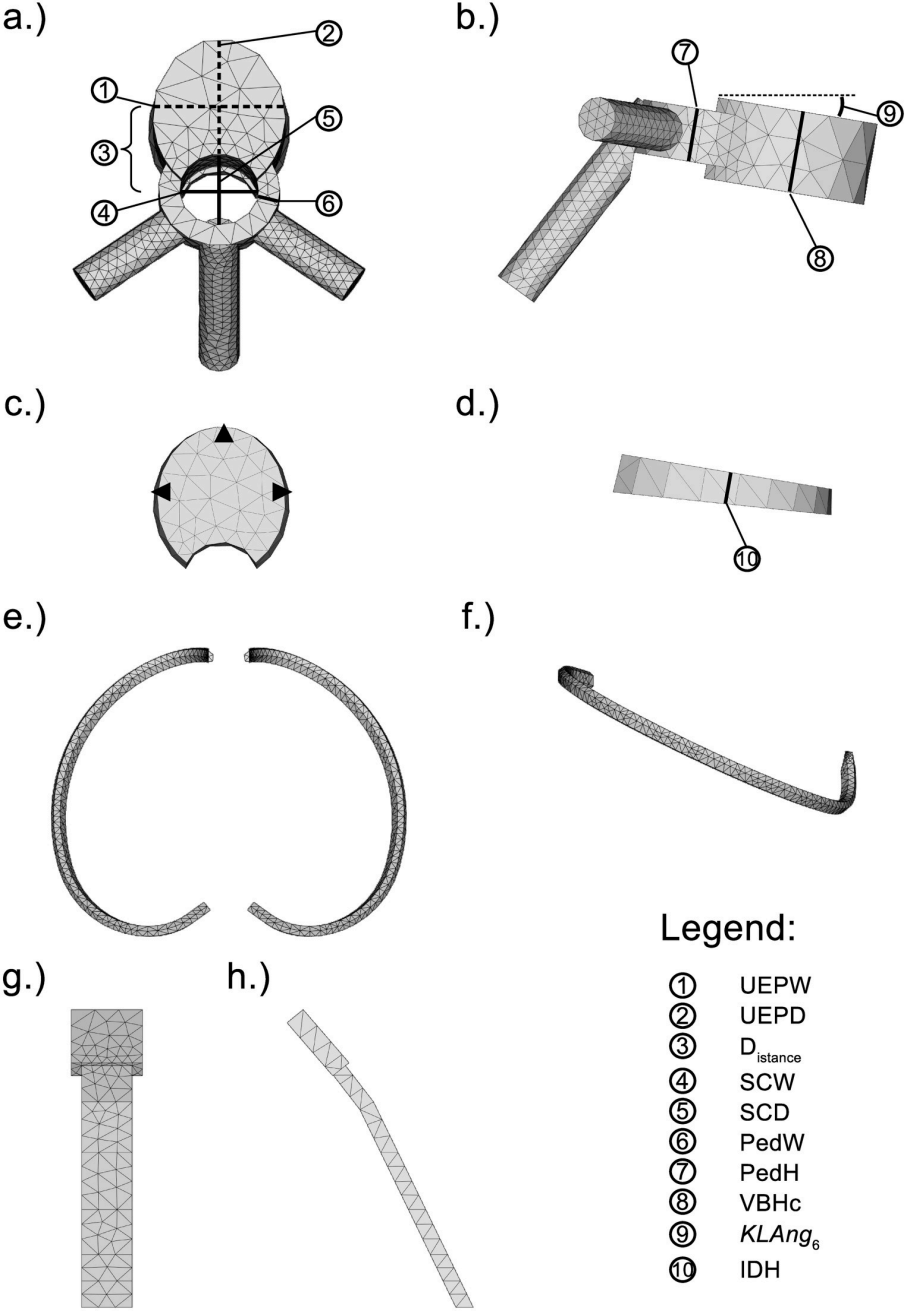
Model components. Principle components used to built the model of the ribcage and vertebral column of a female adult and corresponding anatomical dimension parameters, after [31]. (See this reference also for definition in detail of parameters ①, ②, ④–⑧, ⑩.) a.) and b.): Top and side view of the vertebra T6. c.) and d.): Top and side view of the left and right 6th rib. e.) and f.): Front and side view of the combined manubrium and sternum.

The model of the ribcage and vertebral column (MoRCaVC) was constructed from four components, with individual specifications: 12 thoracic and 5 lumbar vertebral bodies (T1 to L5), 16 IVDs, 2 × 12 ribs (separated into their main body and cartilage part), and the combined manubrium and sternum; see Fig 2. The individual anatomical geometries and morphometric parameters of these components were designed according to literature (given below) and converted into mesh objects with FreeCAD.

**Fig 3:**
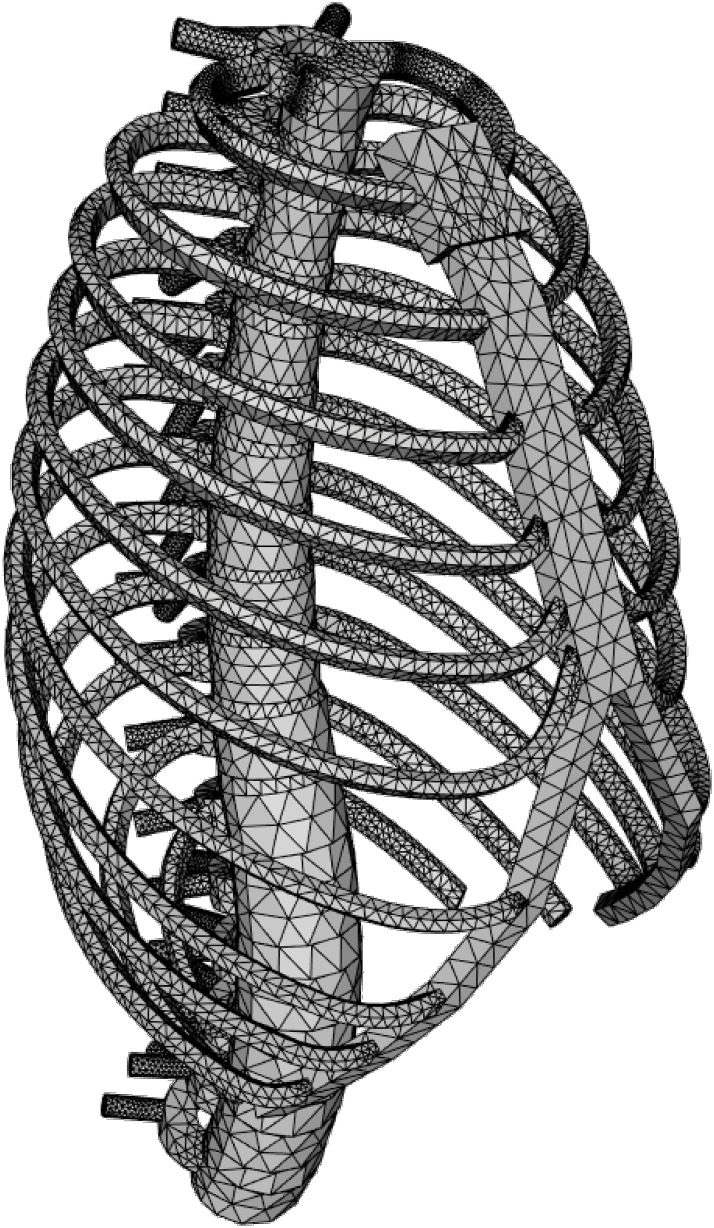
CAD model. Model of ribcage and vertebral column (MoRCaVC) of an adult female, based on anatomical geometry and dimension parameters after [31, 32].

The basic model, shown in Fig 3 from a perspective view, hence represents the MoRCaVC of an average human adult in an upright position. It is gender specific, since in particular the dimensions and geometries of the ribs were defined by different parameters for female and male, after [32]. Furthermore, the model can be scaled along the principle axes to account for the effects of individual body shape and size of the patient. The effect of age can be taken into account by changing the material and structural properties of the components, e.g. the Young’s modulus.

The initial geometries of the vertebral bodies were constructed according to the individual anatomical dimensions of the thoracic and lumbar vertebrae of the human spine, after Busscher et al. [31]. An example model of the thoracic vertebra at T6 level for a female adult is shown in Fig 2 from top (a) and right side (b) together with the corresponding morphometric parameters. For details on acronyms see Table 1 and references therein.

**Table 1:** Acronyms, names and parameters.

Name:	Description:	Source/Reference(s):
3D	Three-dimensional	standard
A	Area	Eq (9)
CAD	Computer-aided design	standard
CP	Cerebral Palsy	standard
CT	Computed tomography	standard
*D*_*istance*_	Longitudinal distance between centres of vertebral body and pedicle arch	intern
FEBio	Software suite	Ref. [35, 36]
FEM	Finite element method	standard
*IDH*	Intervertebral disc height	[31]
IVD(s)	Intervertebral disc(s)	intern
*KAng*	Kyphosis angle	intern
*KLAng*	Vertebra inclination angle	Eq (4)
*KyphR*	Kyphosis radius	Eq (5)
*LAng*	Lordose angle	intern
*LordR*	Lordosis radius	Eq (5)
MoRCaVC	Model of RibCage and Vertebral Column	intern
P1, P2	Patient N°	intern
*PedH*	Pedicle height	[31]
*PedW*	Pedicle width	[31]
*SCD*	Spinal canal depth	[31]
*SCW*	Spinal canal width	[31]
*UEPD*	Upper (vertebral body) end-plate depth	[31]
*UEPW*	Upper (vertebral body) end-plate width	[31]
*VBHc*	Vertebral Body Height central	[31]
*Y*_*Pk*_	Rib aspect ratio	[32]

Acronyms, names and parameters used in the manuscript. The source “intern” refers to a definition in the text.

The basic shapes of the vertebral body and the vertebral arch were considered as full and hollow intersecting elliptic cylinders with individual widths (UEPW), depths (UEPD) and heights (VBHc), after [31]. The longitudinal transverse distances (*D*_*istance*_) between the centres of the intersecting cylinders of the vertebral body and the pedicle arch were estimated in terms of the individual depths of the vertebrae and vertebral arches by:

$$\begin{array}{c} D_{istance}=0.5*\left({UEPD}_{i}+\frac{{SCD}_{i}}{2}*0.8\right). \end{array}$$ (1)

The individual positions of the vertebrae along the vertical body axis—the z-axis—were calculated iteratively according to Eq (2):

$$\begin{array}{c} z_{i}^{\text{VB}}=0.5*\left({\text{VBHc}}_{i}+{\text{VBHc}}_{i+1}\right)+{\text{IDH}}_{i}+z_{i+1}^{\text{VB}}, \end{array}$$ (2)

where the index *i* = 1, …, 12, 13, …, 17, denotes the vertebral level T1, …, T12, L1, …, L5, and $z_{12}^{\text{VB}}=0$. Vertebra T12 was thus located at the origin of the present coordinate system and used as reference point for further calculations. The algorithm and formula presented here, however, do not depend on the choice of origin. That is, any other vertebra can be set as reference point accordingly.

The intervertebral disks (Fig 2c and 2d) were modelled by an elliptic cone segment connecting the adjacent vertebral bodies, with initially individual heights (*IDH*_*i*_), after Busscher et al. [31], and widths and depths according to the adjacent vertebral bodies.

In order to describe the initial, natural S-type shape of the course of the vertebral column in the sagittal plane, offsets along the longitudinal transverse axis—the y-axis—were calculated from:

$$\begin{array}{c} \begin{array}{cc} y_{i}^{\text{VB}}= & (\sqrt{4*\sin{\left(\frac{{\text{KLAng}}_{i}}{2}\right)}^{2}-\sin{\left({\text{KLAng}}_{i}\right)}^{2}}\ldots \\ & \ldots \{\begin{array}{c} +\cos({\text{KLAng}}_{12})-1\\ -\cos({\text{KLAng}}_{12})+1 \end{array})*\{\begin{array}{cc} \text{KyphR}, & \text{for}\;i=1,\ldots ,12\\ \text{LordR}, & \text{for}\;i=13,\ldots ,17, \end{array} \end{array} \end{array}$$ (3)

where

$$\begin{array}{c} {\text{KLAng}}_{i}={\text{KLAng}}_{12}-z_{i}^{\text{VB}}/\{\begin{array}{cc} \text{KyphR}, & \text{for}\;i=1,\ldots ,12\\ \text{LordR}, & \text{for}\;i=13,\ldots ,17 \end{array} \end{array}$$ (4)

and

$$\begin{array}{ccc} \text{KyphR} & = & \frac{|z_{1}^{\text{VB}}-z_{12}^{\text{VB}}|}{\text{KAng}}\\ \text{LordR} & = & \frac{|z_{12}^{\text{VB}}-z_{17}^{\text{VB}}|}{\text{LAng}} \end{array}$$ (5)

with

$$\begin{array}{ccc} \text{KAng} & ≔ & 38^{{}^{\circ}}\\ \text{LAng} & ≔ & 42^{{}^{\circ}} \end{array}$$ (6)

are initial angles similar to the kyphosis and lordosis angles, respectively. According to Eq (5) *KyphR* and *LordR* are the corresponding radii associated with the kyphosis and lordosis angles and hence Eq (4) defines the individual inclination angle around the lateral axis in the transverse plane—the x-axis—of the vertebrae. Initially *KLAng*_12_ was set to 14°.

Further there are no offsets along the lateral transverse axis,

$$\begin{array}{c} x_{i}^{\text{VB}}=0, \end{array}$$ (7)

since the vertebral column is considered to be vertically straight in the coronal plane in its initial state.

The gender specific model ribs were constructed individually for each level, following the morphometrics and equations published by Holcombe et al. [32]. According to them, the individual shape of the in-plane human rib centroidal path at each level was modelled by a parametric curve, built from logarithmic spirals portions for the proximal and distal part of each rib. In total five shape and one size dependent parameters were needed for either rib configuration. That is, each of the twelve pairs of left and right ribs has its individual set of parameters and is different for female and male.

The anatomical out-of-plane “waviness” of the central rib path was not considered though, but could be parametrised and implemented into the present code in a similar manner. The cross sections of the ribs were approximated in the present work by a hexagon, with an transverse out-of-plane length of 12 mm and an in-plane width of 6.8 mm, respectively. The inclination angles of the ribs, that are the angles between the planes spanned by the central rib paths and the y-axis, were set to individual values from 25 to 35 degree [33].

Potential differences between the left and right ribs were not considered here. That is, ribs at the same vertebral level to the left and right at the mid-sagittal plane could be transformed by mirror inversion. An example of the thoracic rib model at level T6 is shown in Fig 2e and 2f from top and side view, respectively.

The combined manubrium and corpus of the sternum was modelled in a rather simple manner by an extruded pentagon and an elongated rectangle, respectively. The corresponding dimensions in lengths and sternum angle for female and male were adapted from the morphometric analysis of the sternum by Selthofer et al. [34]. The centre line of this object was defined relative to the distal endpoints of the thoracic ribs T1 to T10. Its initial position hence changes with variation of the rib shape parameter values. A front and side view of the combined manubrium and corpus of the sternum is shown in Fig 2g and 2h.

### 2.3 FEM calculations

#### 2.3.1 Meshing

In order to analyse the MoRCaVC with FEM, all components of the MoRCaVC were meshed into 4-node linear tetrahedral finite elements, using the in-built tools Netgen Mesher in Mesh FromPartShape in FreeCAD [30]. That is, each 3D component of the MoRCaVC was subdivided into discrete geometric topological cells of triangular pyramid shape with 4 corner points, so-called nodes, for analysis. In a first approach the mesh fineness was set to coarse, in order to keep the number of finite elements small and hence reduce computation time.

#### 2.3.2 Biomechanical properties

The biomechanical features, that are here the material properties and contacts of the components of the developed MoRCaVC, were assigned using a software suite called FEBio [35, 36]. For the present study the vertebrae and the combined manubrium and corpus of the sternum were considered as rigid bodies and thus non deformable objects (ND). The ribs, costal cartilages and IVDs were considered as non-linear elastic, according to a neo-Hookean solid [37, 38]. The corresponding material properties, namely the density, the Young’s modulus and the Poisson’s ratio, of the components are listed in Table 2. The densities were estimated from diverse bone mineral density measurements and were in broad accordance with corresponding parameters used in other FE models [39–42].

**Table 2:** Material properties of the model components. ND: non deformable object; Px: patient number.

Component:	Density (*kg/m*^3^):	Young’s modulus (*GPa*):	Poisson’s ratio:
Cartilage (costal)	1.0 ⋅ 10^3^	10.5 ⋅ 10^−3^ [43]	0.2
Intervertebral disk	1.0 ⋅ 10^3^ [44]	30.9 ⋅ 10^−3^ [45]	0.45 [42, 44]
Manubrium/Sternum	1.0 ⋅ 10^3^	ND	ND
Rib	1.0 ⋅ 10^3^ [39, 40]	11.3 for P1	0.3
		11.7 for P2 [46] (cp. Eq (8)	
Vertebral body	1.1 ⋅ 10^3^ [44]	ND	ND

The Young’s modulus, which is the quotient of uniaxial stress and strain, is a measure of the stiffness of a solid material with respect to its shape. This stiffness varies with age in child rib cortical bone [47, 48] and was therefore considered as a function of age, following the equation:

$$\begin{array}{c} E\left(\text{GPA}\right)=6.71*a^{0.2} \end{array}$$ (8)

where *a* is the age in years, after Zhu et al. [46]. The Young’s modulus of the costal cartilage was set to the average instantaneous elastic modulus value derived from local indentation tests by Forman et al. [43]. The effective modulus of the human IVD, was studied by Yang et al. in cadavers from adults and found to vary considerably with age and strain rate [45]. Since the current MoRCaVC is designed to model adolescent structures, the Young’s modulus of IVD was set accordingly to the measured effective modulus at the low strain rate at the youngest age available, that is 30 years.

In complement the Poisson’s ratio describes the expansion, or contraction, of a material in the directions perpendicular to the applied impact. The given values were set in accordance with literature and other FE calculations on similar problems [42, 44, 49] (and references therein).

In accordance with the material properties of the model components, contacts were set between the vertebral bodies, IVDs, cartilages, ribs and the manubrium/sternum, respectively. Table 3 lists the type of contact between the aforementioned components. Rigid contact therein refers to a contact between a rigid body (here e.g. vertebral body) and a deformable mesh (e.g. intervertebral disk). A tied (node-on-facet) contact connects two non-conforming surface meshes, for instance meshes of elastic components, without sliding across or breaking away from each other [50]. Microscopic biomechanical properties were not considered in the present study.

**Table 3:** Contacts.

Connected elements:			Type:
vertebral body	–	intervertebral disk	rigid
vertebral body	–	rib (proximal end surface)	rigid
rib (distal end surface)	–	cartilage (proximal end surface)	tied
cartilage (distal end surface)	–	manubrium/sternum (lateral surface)	tied

Defined contacts in MoRCaVC, using the nomenclature after [51].

#### 2.3.3 FEM simulation

In order to study the distorted configurations of the spine from the macroscopic structural mechanics of the developed MoRCaVC upon prescribed loads of translation and rotation, FEM simulations were performed using the “solid” module for structural mechanics analysis in FEBio [35, 36]. Initially, at state zero, the vertebral column model was straight in the coronal plane and exhibit the typical S-shape in the mid-sagittal plane from right lateral view. The vertebral bodies at level T1 and L5, that are the top most and bottom most vertebrae of the present model, respectively, were kept fixed in their transverse translational and all rotational degrees of freedom. In addition vertebra L5 was fixed along the z-axis to fix the MoRCaVC in space. All other components of the MoRCaVC were set free and no further boundary conditions or constraints were applied.

In order to simulate a distorted spine, various combinations of lateral displacements and rotations were applied to certain vertebrae between level T1 and L5. That is, each simulation described the resulting effects of a prescribed, successive rigid lateral displacement along the x-axis and/or a rotation about the z-axis to one, or more, vertebral body on the MoRCaVC. All combinations of rigid displacement values, rigid rotation values and vertebral level hence result in a 3D matrix of simulation results with size: *N*_*Displacement*_ × *N*_*Rotation*_ × *N*_*Level*_.

In order to compare the simulation results with the patients data, the former was allowed to undergo a rigid transformation—translation and rotation—and could be scaled along the principle axes to account for the effects of individual body shape and sizes of the patient.

## 3 Results

The resulting models from FEM calculations of the scoliotic spines of two female adolescents were evaluated in combination with the images from a body scanner and spinal X-ray. The 3D scan images were captured by a body scanner, using no ionizing radiation. The model simulations were done in order to analyse the body scanner image and to extract information about the lateral deviation and axial rotation of the vertebrae. In order to describe the analysis procedure all details of the simulation results are showed here at the example of P1. Following the same procedure the simulations were also done for the case of P2. The resulting course of the vertebral column, obtained from the distorted MoRCaVC, is presented and compared with the corresponding course from the X-ray image. In addition stress and strain at the IVDs of the MoRCaVC were analysed as an effect of the scoliotic deformation of the spine for the case of P1.

**Fig 4:**
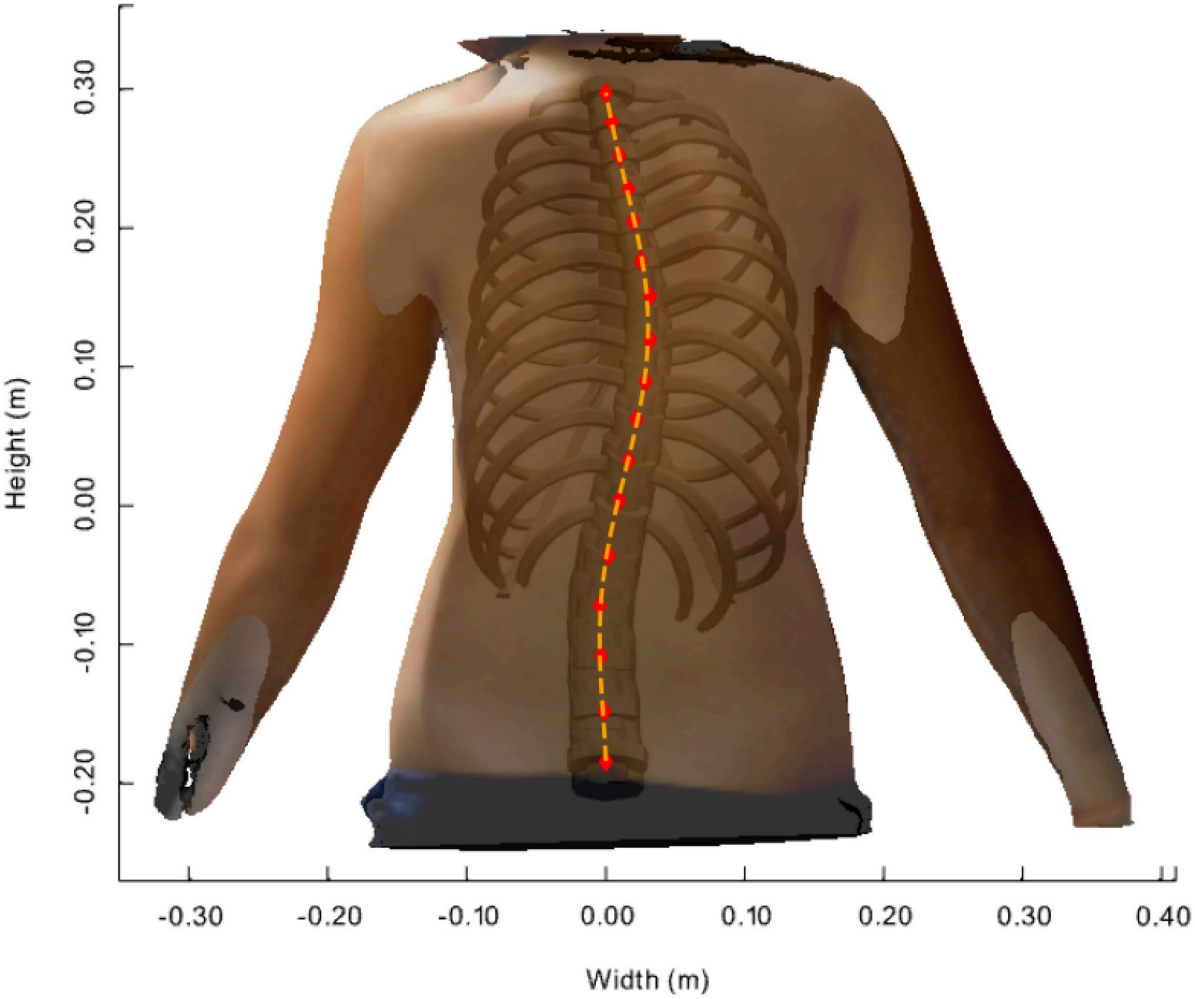
Distorted model inside patient’s body scan image. Transparent back face of an adolescent female 3D body scan with the resulting FEM calculated model inserted at its best match in posterior-anterior view. The markers denote the centre positions of the vertebral bodies along the vertebral column from T1 to L5. The dashed line denotes the course of the distorted vertebral column obtained by least square polynomial fit passing through the marker positions.

Fig 4 shows the posterior-anterior view of the transparent back face of the patient’s (P1’s) body scan image together with the FEM calculation results of the MoRCaVC. A good match between the patient’s body scan and a FEM model calculation was achieved for an initial lateral translation of +30 mm along the x-axis in combination with an effective rotation of -5° around the z-axis at the vertebral level T8 and a small lateral translation in the opposite direction of -5 mm at the vertebra L2 with respect to their initial positions and orientation. In parallel the initial rib aspect ratio (*Y*_*Pk*_), one of the five rib shape parameters that defines the ratio between length and peak height of each rib (after [32]), had to be slightly increased, in accordance with the findings and the variability observed and associated with the effects of age and demographics on the rib shape by Holcombe et al. [52]. Also, the model had to be scaled by factors 1: 0.93: 1.12 along the principle axes *x*: *y*: *z*, respectively, to account for the patient’s body size. After getting a good match between the patient’s body scan and the FEM model, the positions of the centres of the vertebral bodies from T1 to L5 were located (red markers in Fig 4) along the vertebral column using the analysis tools described in [29]. A polynomial was then used to fit a curve passing through these points, i.e. the positions of the vertebral bodies (orange dashed line in Fig 4). Therefore, this curve describes the course of vertebral column in the coronal plane obtained from distorted MoRCaVC which is expected to coincide with the trajectory of the patients spine as seen in the scan image.

**Fig 5:**
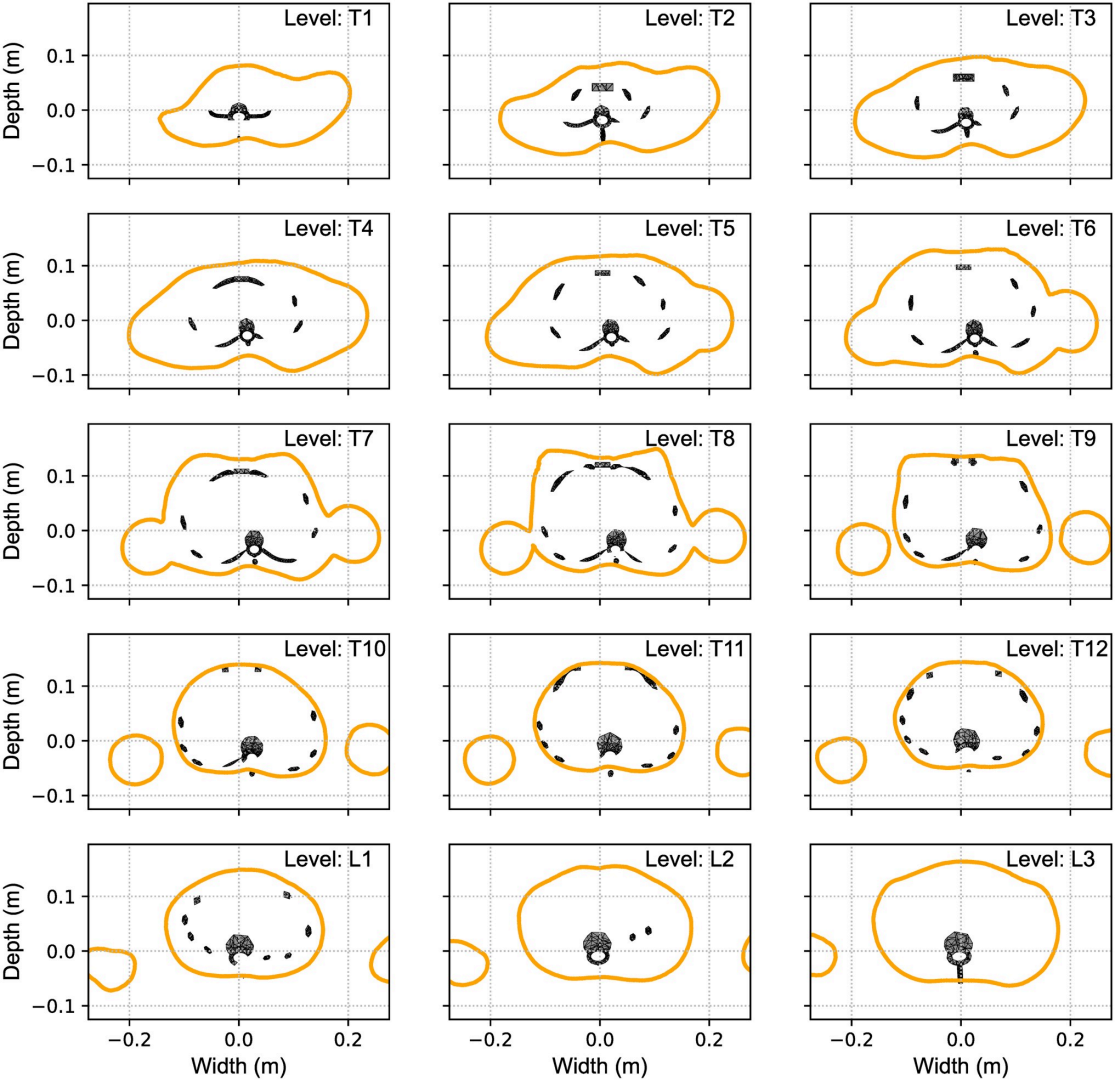
Transverse matching contours. Transverse cuts at the vertebral levels T1 to L3 through the body scan image (shown in Fig 4) with the FEM calculated model inserted visualizing the individual lateral translation and axial rotation of the vertebrae and ribcage at its best match.

The corresponding transverse cuts of the body scanner image with the results of the FEM calculated MoRcaVC inserted are shown in Fig 5 for the vertebral levels T1 to L3 for the the case of P1. Each subplot in Fig 5 shows for each vertebral level the transverse contour line of the 3D body scan image (solid contour line) in relation to the distorted ribcage geometry (inset). This also visualises the resulting individual lateral translation and axial rotation of the vertebrae and attached ribs. As mentioned afore, a lateral translation and rotation of the vertebra T8 in combination with a small opposite lateral translation of vertebra L2 were necessary to achieve the good match between the model and the scanned image.

**Fig 6:**
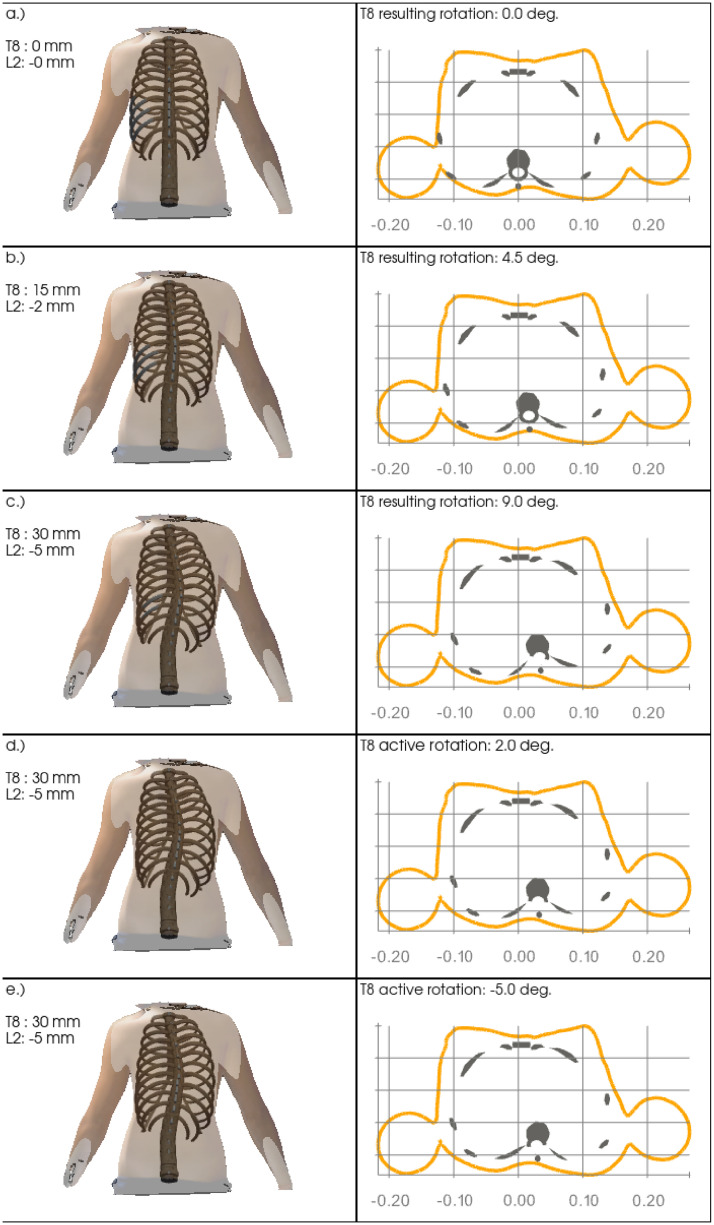
Sequential decomposition. Sequential model distortion in relation to the static body scan image upon opposite lateral translations at the vertebrae T8 and L2 (panels a.) to c.) and subsequent active clockwise axial rotation of vertebra T8 (panels d.) and e.)). While the coronal views (left panels) visualize the overall lateral model distortion, the resulting anti-clockwise rotation of vertebral T8 from only lateral translations at the vertebrae T8 and L2 is visible in the transverse views (right panels).

Fig 6 shows the model response and effective results upon a lateral translation of the vertebrae T8 and L2 and rotation of the vertebra T8 in sequence, in order to assess the relation between lateral translation and axial rotation at the most affected vertebra. Left panels show the simulation results and the transparent body scan image in posterior-anterior view from initial (top) to final (bottom) state. Accordingly right panels show the corresponding transverse sectional views at the vertebral level T8. The values of lateral translations and effective rotations at vertebra T8 are given in the annotations.

Subplots a.) to c.) show the effective response of the model upon pure lateral translation of +0, +15, +30 mm and -0, -2, -5 mm of the vertebrae T8 and L2, respectively. While the lateral deviation can be clearly seen in the posterior-anterior coronal view and the transverse cuts, the latter also clearly show a rotation of the vertebra towards the concave side of about +9° at its maximum at level T8, see Fig 6c) (right column). Subplots d.) and e.) then show the resulting effects of the additional subsequent rotation of about -14°, from +9° to -5°, around the z-axis of the vertebra T8, yielding a small rib hump on the convex side, visible in the transverse cut.

**Fig 7:**
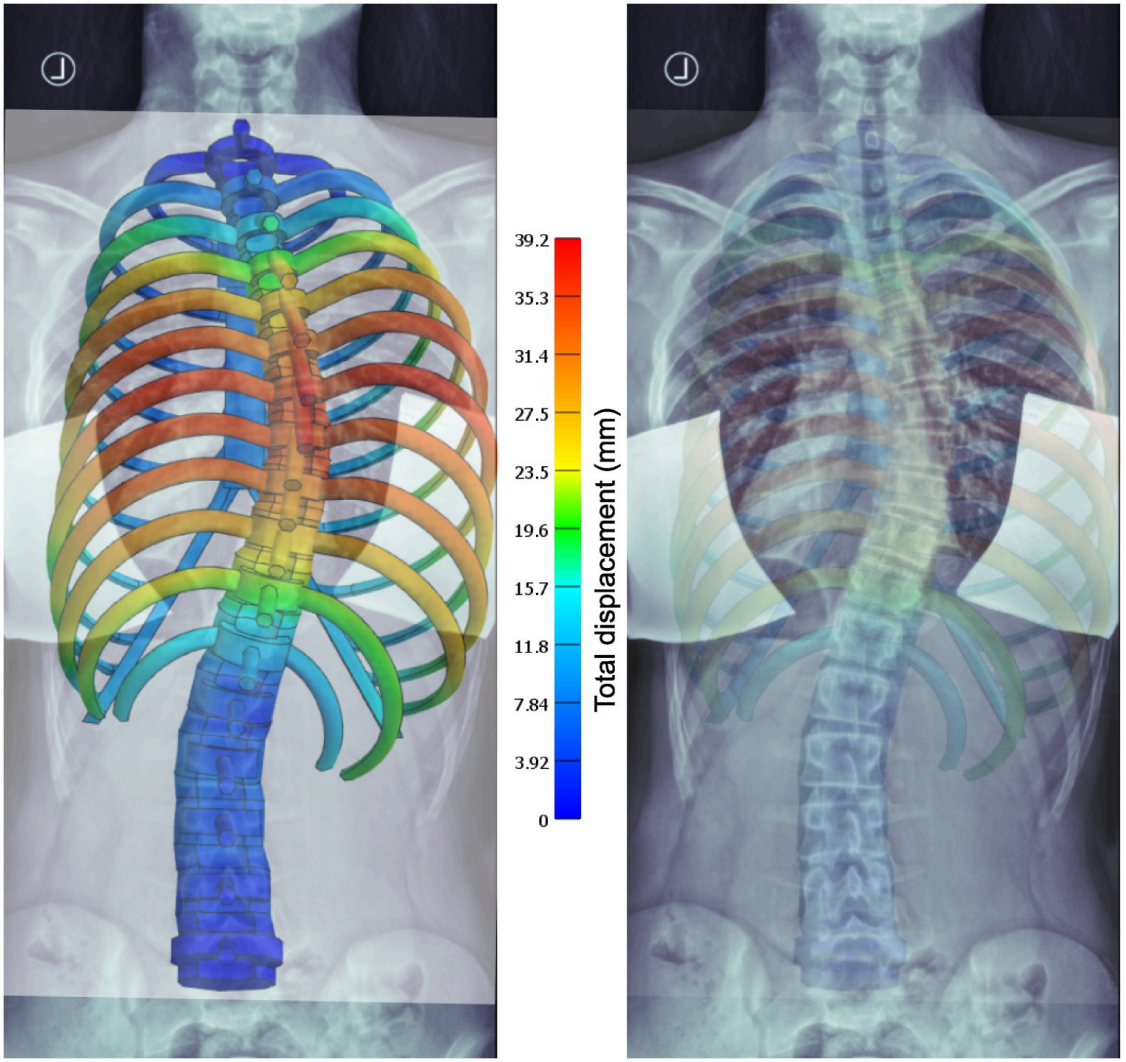
Comparison with X-ray. Overlay of X-ray and simulation result images at best match according to visual inspection, with different transparencies from left to right (for better visualization). The colour code shows the total displacement from the initial state—a straight vertebral column in the coronal view.

A comparison of the FEM calculation results with the X-ray image of the patient P1 is shown in Fig 7. For best matching initial lateral translations of +33 mm instead of +30 mm at T8 and -5.5 instead of -5 mm at L2, respectively, were necessary. The graphical overlay shows a good match between the course of the vertebral column and the simulation and a reasonable representation of the outer ribcage contour at the right convex side, while the simulation does not fully represent the outer ribcage contour at the concave side.

**Fig 8:**
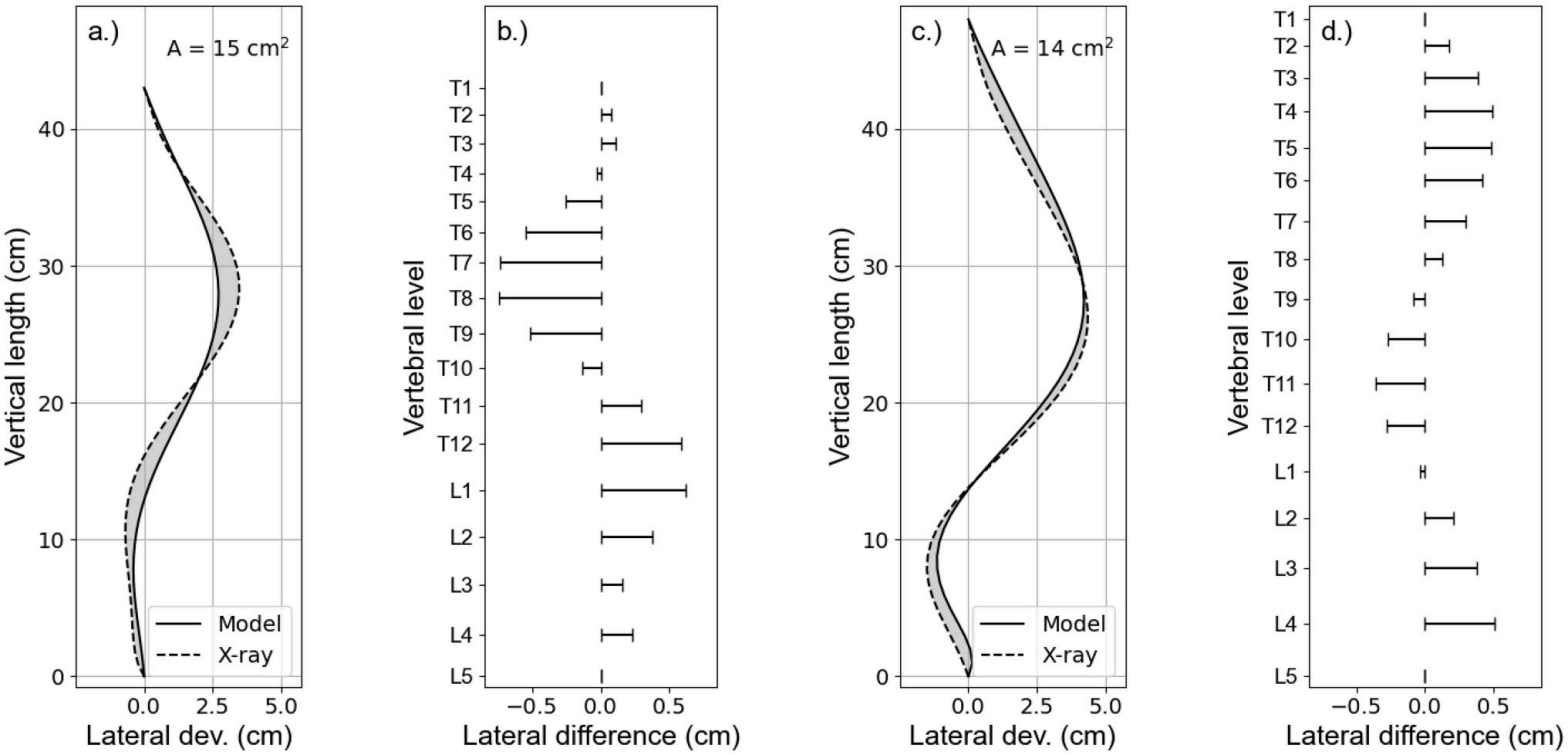
Model vs. X-ray. Comparison of the courses of the vertebral column extracted from X-ray (dashed lines) and model calculation (solid lines) for patients P1 and P2 (a.) and c.)), respectively, showing their absolute lateral deviation. The absolute lateral differences between the curves at the vertebral levels for either patient are depicted in b.) and c.). The parameter A quantifies the grey area between the curves and hence represents a measure for the degree the courses derived from X-ray and model calculation matches each other. (Note that the X-axes have been scaled for better visualization).

Fig 8 shows overlays of the polynomial fits, representing the courses of the vertebral columns in the coronal plane, derived from X-rays (dashed lines) and model simulations (solid lines) for the two patients P1 and P2, respectively (a.) and c.)). The polynomials were obtained by fitting the locations of the centres of vertebral bodies (Figs 1 and 4). The two polynomials of either patient were normalized to their vertical length between the vertebral levels T1 and L5 and aligned to coincide at the top and bottom most vertebrae T1 and L5. The gray area between the lines is a measure for the total difference between the curves. It has been calculated following the equation:

$$\begin{array}{c} A\left(x,y\right)=\lim_{N\to \infty }\sum_{i=0}^{N-1}|x_{i}^{X-ray}-x_{i}^{mod}\left|\cdot \right|y_{i}-y_{i+1}| \end{array}$$ (9)

where *x*^*X*−*ray*^ and *x*^*mod*^ are the x-coordinates on the polynomials derived from X-ray and the distorted model, and *y*_*i*_ are the corresponding y-coordinates. The latter have identical values for X-ray and the distorted model. The absolute lateral differences at each vertebral level are depicted in details in Fig 8b and 8d. The calculated values obtained from Eq (9) are indicated in the Fig 8 for either patient.

**Fig 9:**
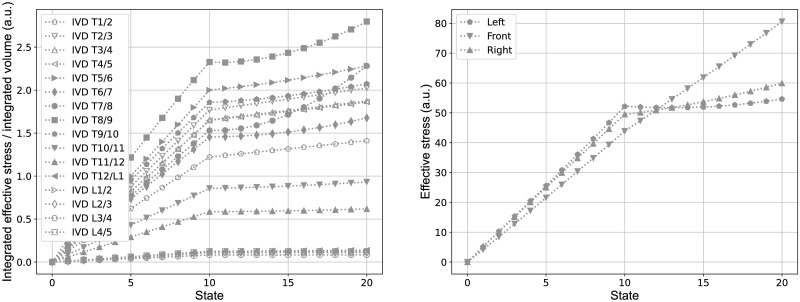
Stress on intervertebral discs. Effective stress integrated per volume of all IVDs (left) and at three principle nodes in the transverse plane between the vertebral level T8 and T9 (right). The states zero to ten correspond to the prescribed lateral displacements at the vertebral levels T8 and L2 and the states eleven to twenty correspond to the subsequent rotation around the z-axis of the vertebra T8, in accordance with the sequential representation in Fig 6.

Besides the changes in macroscopic structural mechanics and the response of MoRCaVC, stress and strain at the IVDs were extracted from the FEM calculations in order to see their effect in context of spinal deformations. Fig 9 shows the effective stress in the IVDs evolving with the lateral displacements of the vertebrae T8 and L2 (state zero to ten) and the subsequent rotation of the vertebra T8 (state eleven to twenty), in accordance with the sequence shown in Fig 6 where the subplots a.) to e.) correspond to the present states 0, 5, 10, 15 and 20, respectively. The integrated effective stress normalized to the integrated volume, hence, increases linearly with the lateral displacement and with higher gradient around the apex level at T8, where the effective total displacement is maximal. The subsequent rotation shows a further increase of the integrated effective stress, but with smaller gradient. Moreover, the integrated effective stress values are higher for IVDs at levels in the thoracic range than in the thoraco-lumbar and lumbar range.

The effective stress, however, is not equally distributed in the transverse plane of the IVDs, as shown at the example of the level T8/9, at three specific nodes at the left, front and right side, marked in Fig 2c. The data show a larger gradient of the effective stress at the left and right side in comparison to the front side upon the lateral displacement (state zero to ten) and an inverse trend for the vertebra rotation (state eleven to twenty). That is there is a larger stress increase at the lateral sides upon the displacement and a larger increase at the anterior side with rotation.

**Fig 10:**
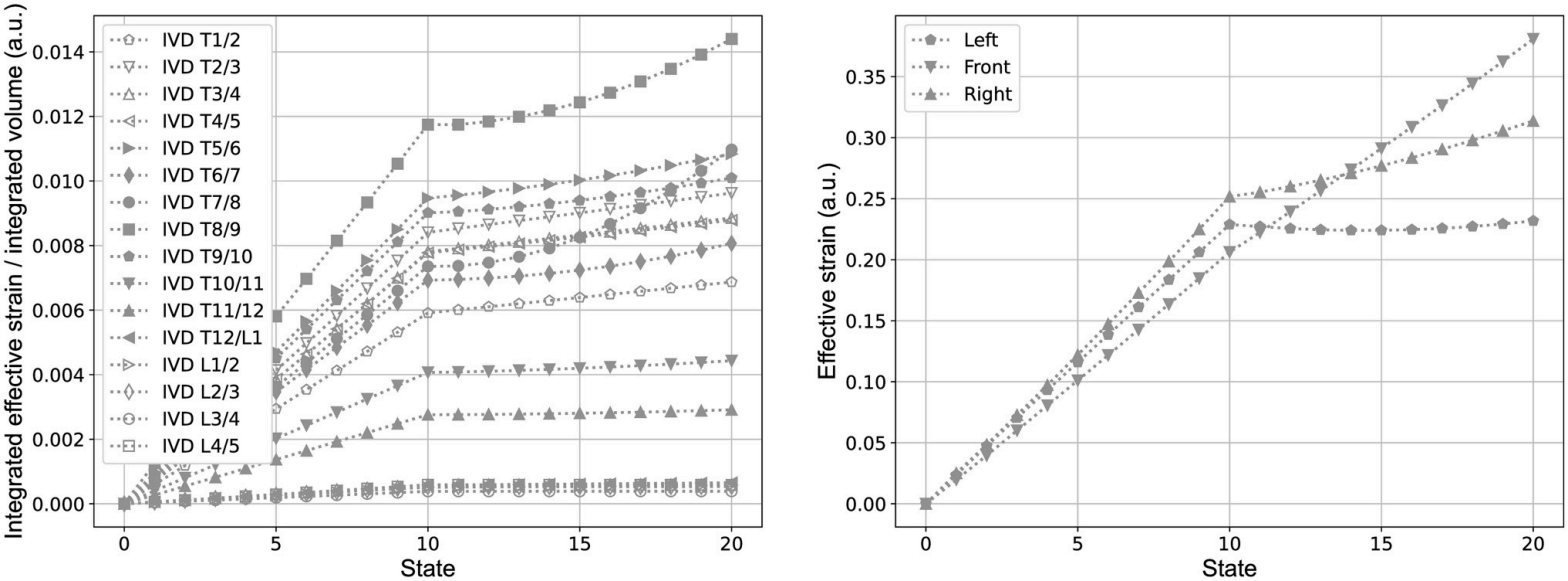
Strain on intervertebral discs. Effective Lagrange strain integrated per volume of all IVDs (left) and at three principle nodes in the transverse plane between the vertebral level T8 and T9 (right). The states zero to ten correspond to the predefined lateral displacements at the vertebral levels T8 and L2 and the states eleven to twenty correspond to the subsequent rotation around the z-axis of the vertebra T8, in accordance with the sequential representation in Fig 6.

Similarly Fig 10 shows the variation of the effective Lagrange strain per volume at the same intervertebral disc, due to the lateral displacements of the vertebrae T8 and L2 (state 0 to 10) and the subsequent rotation of the vertebra T8 (state 11 to 20). The integrated effective Lagrange strains, hence, show similar trends then observed for the integrated effective stress. The effective Lagrange strain at the specific nodes, described afore, show in principle similar trends to the effective stress at these points, but with a clear difference between the left and right side. Here the latter, that is the convex side, shows larger effective strain values than at the concave/left side. The reason for this effect, similarly seen also at other IVDs, is subject to further investigation by now.

## 4 Discussion

FEM based biomechanical simulations on a self-developed ribcage model were used to evaluate scoliotic spine. The model and analysis were developed with the ultimate aim of estimating spinal deformations from 3D surface scan images of patient’s torsos, captured with a body scanner free of ionizing radiation. The simulations provide a broad view of the lateral translations and axial rotations of the vertebrae and the distribution of stresses and strains on the IVDs, due to the mechanical distortion. This might help to further understand the pathogenesis of scoliosis, which is of particular avail for adolescents. The courses of the vertebral column derived from the X-ray image and from the ribcage model, which is expected to coincide with the spinal trajectory in the scan image, were stacked to show their degree of matching. Thereby the results obtained from the body scanner are compared with a conventional X-ray method. In a first approach, the developed model presented here was applied to two adolescent females with scoliosis.

Reasonable good matchings of the present model with the 3D body scan (Figs 4 and 5) and the patient’s X-ray image (Fig 7), respectively, were obtained by visual inspection. Since the assessment of the course of the spine by scanning methods is in general limited, we have intentionally combined in our method the three-dimensional scan image with simulations of the bony spine model. Our new approach therefore cannot be compared with the limitations of general surface scan methods. The small difference in lateral translation in comparison of the matchings of the 3D body scan and the X-ray image with the model simulation results might be due to unintentionally slightly different standing positions of the patient during either measurements. Further adjustments to the kyphose angle might potentially improve the results at the upper end of the vertebral column, but would have no relevant effect on the distortion of the vertebrae around the apex level. The effects of age on the shape, position and structural properties of the ribs [52, 53], and the impact of ligaments, musculature and soft tissue [54, 55] on the biomechanics, will be subject of further improvements. Thus natural growth, posture, body fat etc. are not considered in the present study. The starting configuration and some of the material properties of the present model (e.g. Young’s modulus of IVDs), however, are based on an average human anatomy. Further tests on a larger set of data are hence necessary to prove its applicability on adolescent idiopathic scoliosis.

In our previous work, we have shown that different shapes of the two dimensional transverse cross sections of the torso through asymmetry parameters, could capture the intra- and inter-individual characteristics associated with scoliosis [56]. Particularly, in case of mild and moderate scoliosis the analysis method delivered good estimates of the vertebral column trajectory [29]. These studies were based on computed tomography (CT) images, which incidentally showed scoliosis. The CT scans, hence, were not performed purposely for these studies, but collected from the pool of available images of patients that attended the hospital for other reasons. The present study showed that additional information about the position, axial rotation and inclination of the vertebral bodies at all thoracic and lumbar vertebral levels can be extracted from the two dimensional transverse cuts by FEM model calculations.

The present simulation results suggest that a lateral translation of the vertebral body at level T8 results in an effective axial rotation towards the concave side of the spinal curvature in coronal view (Fig 6). An additional active rotation of the vertebral body in the opposite direction, however, could reproduce the rib hump at the convex side seen in the transverse cross section of the body contour. Further analysis of the FEM calculation results showed that a simple lateral translation at the vertebral level T8 by itself is not sufficient to represent the patients body contours. A small lateral translation in the opposite direction at the vertebral level L2 was necessary in order to keep the lumbar vertebral column rather straight and represent the marginal left convex balancing curvature. Simulations without these counter translation did show a rather large C-shaped vertebral column between the levels T1 and L5 and did not sufficiently match the patients scan. Pure lateral translations at the vertebral levels T8 and L2, did result in an effective rotation of the vertebral column around the z-axis, yielding a rib hump on the concave side of the main curvature. An opposite rotation of the vertebra T8, hence, was necessary in order to achieve the afore described results. Furthermore, a good agreement was obtained between the courses of the vertebral column extracted from the X-ray image and the scan image, with the help of model simulations, for both patients (Fig 8). The quality of the matches were quantified by the area A between the curves. That is, A is a quantification of the degree the course of the vertebral column from the model matches the patients anatomy of the course of the vertebral column seen in the X-ray.

The present combined method of 3D surface body scan and FEM model simulations, hence, provide quantitative anatomical information about the position, rotation and inclination of all thoracic and lumbar vertebrae, which are important in the assessment of scoliosis. And thus brings our methods closer to being implemented in clinical practice. In addition, the flexibility of the MoRCaVC simulations to account for individual body shapes and to adapt to the subject specific course of spine might help to gain further understanding of the pathomechanism of scoliosis.

The present results also support the fact that scoliosis is associated with lateral translation and rotation of the vertebrae. Cause and effect, however, are widely discussed. Some studies suggested that an impaired disproportionate growth of the anterior and posterior elements of the vertebrae in children and adolescents might contribute to the translation and rotation of vertebrae [57–59]. In complement, Stokes et al. discussed biomechanical calculations, which indicate that an disparate growth of vertebral bodies in adolescents result from an imbalance in muscular loading and thereby lead to a progression in scoliosis [60]. Furthermore, congenital abnormalities of the vertebral cartilage have also been described as a cause of abnormal vertebral body development during growth [61, 62]. A developing scoliosis therefore results in an disparate growth of the vertebrae and in consequence to a deteriorated degree of scoliosis [60].

The distribution of stress and strain within the IVDs and across different levels, shown in the present study (Figs 9 and 10), indicates that the effective stresses and strains are predominantly associated with the lateral displacement of the vertebral column, but are also influenced by rotation. The overall trend that the integrated effective stresses and strains are larger in the thoracic region than in the thoraco-lumbar and lumbar range might be due to the fact that the adjacent vertebral bodies to the IVDs in the thoracic range are linked with the rigid combined manubrium and sternum, while in the thoraco-lumbar and lumbar range the adjacent vertebral bodies are not constrained in this manner. The slightly elevated values of them at the IVDs T1/2 and T2/T3 might be due to the additional constraints of vertebra T1, which was fixed in the transverse plane and in rotation in order to keep the superior and inferior end of the vertebral column in place.

The evidence of unequally distributed stresses and strains across the IVDs in a scoliotic spine, observed here, are in line with other studies [63], which also found significantly different stress-strain profiles between patients with scoliosis and control groups, as well as between the concave and convex side [64]. With increasing age, various complications can occur in patients with scoliosis, where the malpositions of the vertebra bodies can be associated with damage of the spinal discs and the intervertebral discs. Our considerations of stress and strain in the model can accordingly help to better understand and calculate the biomechanical incorrect loads that affect the intervertebral discs.

The present elementary model in general supports the basic concepts associated with scoliosis. Improvements of the model and extensive tests on a large set of data are thus necessary, before its application in clinical practice as a supplementary tool to conventional examination methods. Techniques free of ionizing radiation, like the present method, however, cannot replace X-ray and other radiographic examinations in the medical assessment of scoliosis. In particular, if surgical intervention is considered, conventional methods based on X-ray and MRI are indispensable to gain precise subject specific anatomical information. In combination with clinical examinations the present method, however, has the potential to reduce the number of X-rays during follow-up scans. Further, the present self-developed model enables various configurations of the vertebral column and ribcage to be simulated in order to further investigate the biomechanics and pathomechanisms associated with scoliosis. These configuration simulations also have the potential to be used for an automatic analysis method in future scoliosis assessment, based on machine learning algorithms, which require analysis of large sample data sets though. The present work, here described, thus constitutes a preliminary step in this direction.
